# Effects of *Penicillium chrysogenum*-Fermented Cotton Straw on Lactation Performance, Serum Biochemical Parameters, and Rumen Microbiota in Saanen Dairy Goats

**DOI:** 10.3390/microorganisms14102133

**Published:** 2026-09-22

**Authors:** Kailibinuer Abudukaiyoumu, Shuai Fang, Yuxia Yang, Changjiang Zang, Jiaojiao Gao, Yuanyang Yi, Yanjie Tai, Tongjun Guo

**Affiliations:** 1 Feed Research Institute of the Xinjiang Uygur Autonomous Region Academy of Animal Science, Urumqi 830011, China; 17767554842@163.com (K.A.); fangshuai1011@139.com (S.F.); gaojiaojiao@163.com (J.G.); 15022970169@163.com (Y.Y.); 2 Xinjiang Key Laboratory of Herbivorous Livestock Feed Biotechnology, Urumqi 830011, China; 3 College of Animal Sciences, Xinjiang Agricultural University, Urumqi 830052, China; zcj780@126.com; 4 Xinjiang Nala Benyuan Animal Husbandry Co., Ltd., Yining 835401, China; lzh130227@163.com

**Keywords:** *Penicillium chrysogenum*, cotton straw, Saanen dairy goat, safety evaluation, lactation performance, biochemical indicators, rumen fermentation, microbiota

## Abstract

Cotton straw, a major agricultural residue in China, is underutilized for ruminant feed owing to its recalcitrant lignin barrier, which impairs palatability and in vivo digestibil-ity. Solid-state fermentation using ligninolytic fungi provides a sustainable strategy to upgrade this lignocellulosic biomass. Nevertheless, the physiological outcomes of feeding fermented cotton straw to lactating ruminants remain largely unexplored. Herein, we investigated the effects of cotton straw fermented by *Penicillium chrysogenum* YL-11 on milk production, serum biochemical profiles, and ruminal microbial community composition in Saanen dairy goats, together with a biosafety assessment of the strain using a murine model. A completely randomized design was employed, involving 30 Saanen dairy goats (2–3 parities, initial body weight 48.56 ± 4.74 kg, milk yield 1.03 ± 0.27 kg/d, days in milk 180 ± 5.82 d) randomly assigned to three groups (*n* = 10): control (CK1, no cotton straw), CK2 (20% unfermented cotton straw), and YL-11 (20% fermented cotton straw). The trial included a 7-day adaptation and a 60-day experimental period. The results demonstrated that YL-11 administration did not adversely affect organ indices, serum biochemistry, or immune parameters in mice (*p* > 0.05). In dairy goats, YL-11 significantly increased average daily milk yield and 4% fat-corrected milk yield (*p* < 0.01), while the feed-to-milk ratio was significantly lower than that of the CK2 group (*p* < 0.01), but did not differ significantly from the CK1 group. Apparent digestibility of crude protein and ash was significantly higher in the YL-11 group than in the CK2 group (*p* < 0.05), with no significant differences observed between the YL-11 and CK1 groups. Milk fat and lactose percentages were also increased in the YL-11 group compared with both control groups (*p* < 0.05). Antioxidant enzyme activities (T-AOC, SOD, CAT, GSH-PX) were elevated, whereas MDA, TNF-α, IL-1β, and TGF-β1 were decreased (*p* < 0.05). Rumen ammonia nitrogen and the acetate/propionate ratio were increased significantly (*p* < 0.05) and the abundance of fiber-degrading taxa, including *Verrucomicrobiota* was enriched in the YL-11 group, with five potential biomarkers identified by LEfSe analysis. In conclusion, *P.chrysogenum* YL-11 is safe and its fermented cotton straw improves lactation performance, rumen fermentation, antioxidant status, and fiber-degrading microbiota in dairy goats, offering a promising strategy for utilizing cotton straw as a feed resource.

## 1. Introduction

Cotton straw, a major by-product generated during cotton processing, with an an-nual output of approximately 30 million tons in China [1,2], is a widely available and low-cost agricultural residue with great potential for feed utilization. It can serve as a roughage source for ruminants; however, its high lignin content [3] results in poor palatability and low digestibility, thereby restricting its large-scale application in livestock production [4,5].

Microbial solid-state fermentation is an efficient and environmentally friendly technical way to degrade lignocellulose in straw and improve the feeding value of straw [6]. A large number of studies have confirmed that straw fermentation by functional strains such as white-rot fungi, Bacillus and yeast can specifically break the lignin structural barrier, release usable carbohydrates, improve substrate crude protein content and in vitro fermentation characteristics, and then improve ruminants’ performance and body health [7]. Zhao et al. [8] use lignin-degrading fungi to treat corn stover, effectively reduce lignin content, improve the nutritional structure of straw, and then optimize its ruminal fermentation characteristics, which can improve the feed intake and milk yield of dairy goats, and enhance the feeding value and market competitiveness of goat milk. Wu et al. [9] added *Saccharomyces cerevisiae* XR4 and *Kluyveromyces marxianus* BC strains to the dairy goat diet, a forage mixed diet at a ratio of 1:1, which could effectively alleviate mastitis induced by a high-concentrate diet. It can improve the health of mammary glands by regulating rumen flora and reducing inflammation and oxidative stress. Li et al. [10] added Bacillus subtilis, Lactobacillus reuteri and Saccharomyces cerevisiae into the diet of dairy goats, adding it to co-fermented silymarin powder, which improved the yield and antioxidant properties of dairy goats and inhibited methanogens, indicating that the feed conversion rate was improved and methane emissions could be reduced. Although a lot of research progress has been made in straw fermentation technology, the existing studies mostly focus on substrates such as corn straw and traditional Chinese medicine by-products, and there is still a lack of in vivo systematic evaluation of lactating ruminants using specific and efficient degrading strains of local cotton straw in Xinjiang. Most studies only focus on the changes in the nutritional composition of straw before and after fermentation, lacking complete feeding test data for lactating animals, and there is insufficient multi-dimensional joint analysis of milk production performance, milk quality, serum biochemistry, ruminal fermentation characteristics and rumen microbiota. Moreover, the internal mechanism of fungal fermentation of cotton straw in regulating ruminal fiber-degrading flora has not been clarified [11,12]. The Saanen dairy goat is the leading breed of dairy goat breeding in Xinjiang, which has strong adaptability, stable lactation performance and excellent milk quality. It is an important basis for the development of a characteristic dairy industry and the income increase for farmers and herdsmen [13]. Over the past few years, the scale of the dairy goat industry in Xinjiang has continued to expand, but the shortage of high-quality alfalfa, oat grass and other coarse feedstocks and the high cost of outsourcing have become the core bottleneck for improving the quality and efficiency of the industry. The development of cotton straw resources with abundant local reserves and the replacement of some high-quality forage grass after microbial fermentation can effectively reduce the diet cost and improve breeding efficiency, which has important practical significance and industrial value for promoting the high-quality development of the Xinjiang dairy goat industry, helping rural vitalization and stable income increase of farmers and herdsmen [14].

In our previous study, we isolated a highly efficient lignin-degrading strain YL-11 from the decaying wood of *Populus euphratica* Oliv and the surrounding soil in Xinjiang, and identified it as *Penicillium chrysogenum*. The degradation rate of lignin in cotton straw reached 36.05%, indicating a good application prospect. Whether this strain can safely and efficiently ferment cotton straw feed and improve the performance and body health of dairy goats in actual production remains to be elucidated. Therefore, this study was designed to assess the feeding effect of cotton straw fermented by *Penicillium chrysogenum* on dairy goats, in order to provide a technical basis and reference for resource utilization of cotton straw.

## 2. Materials and Methods

### 2.1. Safety Evaluation Study Design for the Test Strain

Test strain: The tested strain was *Penicillium chrysogenum* YL-11, which has been deposited at the China Center for Type Culture Collection (CCTCC, Wuhan University, Wuhan, China), with the accession number CCTCC M 20261726. Potato dextrose agar (Haobao Biotechnology, Qingdao, China) was used for culturing at 28 °C for 3–5 days to prepare the bacterial suspension with a viable bacterial concentration of 1 × 10^9^ CFU/mL for later use.

Experimental animals: One hundred and twenty specific pathogen-free (SPF) grade male Kunming mice were sourced from the Animal Experiment Center at Xinjiang Medical University, Urumqi, China, with an age of (5 ± 1) weeks and a body weight of (20 ± 2) g, were selected and housed in an SPF-standard laboratory animal room. After 7 days of acclimatization, the mice were randomly divided into two groups: the CK group (treated with 0.5 mL of 0.9% saline) and the YL-11 group (treated with 0.5 mL of 1 × 10^9^ CFU/mL *Penicillium chrysogenum* YL-11 bacterial suspension per mouse per day by oral gavage), with 60 mice per group. Each group contained six biological replicates, each replicate contained 10 mice. All mice were fed a standard basal diet (SPF rat breeding feed, purchased from Henan Huanyu Hekang Biotechnology Co., Ltd., Anyang, China) and were given ad libitum access to food and water throughout the study period. The animal room was maintained under controlled conditions at 25 °C, with a relative humidity of approximately 60% and a 12 h light/12 h dark photoperiod. The total experimental period lasted 21 days, consisting of a 7-day pre-test acclimation phase followed by a 14-day formal testing phase.

This experiment was approved by the Experimental Animal Ethics Committee of the Feed Research Institute, Xinjiang Uygur Autonomous Region Academy of Animal Sciences, approved this experiment, Xinjiang Uygur Autonomous Region Academy of Animal Sciences (authorization number: a2025-1206). Throughout the experiment, international ethical guidelines for the welfare of experimental animals were strictly observed. Animal housing standards were met, adequate food and water were provided, and stress and injury were minimized through standardized procedures.

### 2.2. Preparation of Fermented Cotton Straw

Cotton straw used in the experiment was provided by Xinjiang Xuze Biotechnology Co., LTD. After cutting to 2–3 cm in length, it was pretreated with a steam blasting device (H-se-01, Xinjiang Hongyirun Biotechnology Co., LTD., Urumqi, China). Subsequently, the pretreated straw was transferred to a baling machine (RX-DK5252C, Shandong Leling Ruixiang Machinery Co., LTD., Leling, China) by a conveyor for baling and pressed into cylindrical parcels with a height of about 0.6 m and a diameter of about 0.6 m, each weighing about 50 kg. The outer layer of the wrap was wrapped with 6 layers of stretch film (MY100, Tianjin Zeheng Packaging Products Co., LTD., Tianjin, China; width 250 cm, thickness 25 μm). The packaged wrap was stored in a well-drained warehouse and used for animal experiments after 45 days of fermentation.

The specific steps are as follows: First, dissolve 150.0 kg sugarcane molasses (7.5% of the substrate dry matter) in 300.0 L water, add 60.0 L *Penicillium chrysogenum* YL-11(CCTCC M 20261726) bacterial solution (3.0% of the substrate dry matter; 6.0 L of bacterial stock solution was mixed with 54.0 L warm water at 20 °C and left for 30 min at room temperature for later use), mix well, spray evenly onto 2 t of steam-exploded cotton straw in layers, and then spray 2973 L water. The moisture content was adjusted to 65%; subsequently, the material was sealed and subjected to fermentation for 45 days. At the end of fermentation, the nutrient composition and free gossypol (FG) content of the fermented product were measured. The concentrations of trimethoprim and patulin were detected by Wuhan Savel Biotechnology Co., LTD, for safety evaluation. The nutritional level, FG content and trimethoprim and patulin test results of the cotton straw and fermented cotton straw are presented in Table 1.

### 2.3. Design and Management of Animal Experiments

The Science and Technology Ethics Committee of the Academy of Animal Sciences of Xinjiang Uygur Autonomous Region reviewed and approved the animal treatment in this study (approval no. 1120241220).

The experiment was carried out from 4 July 2025 to 9 September 2025 in Nala dairy goat farm, located in Gongliu County of Yili Kazakh Autonomous Prefecture, Xinjiang Uygur Autonomous Region. Thirty healthy Saanen dairy goats with similar body condition, 2–3 births, body weight of (48.72 ± 4.74) kg, milk yield of (1.03 ± 0.27) kg/d, and lactation days of (180 ± 5.82) d were randomly divided into 3 groups with 10 replicates per group and 1 dairy goat per replicate. The experiment consisted of a 7-day pre-test period and a 60-day experimental period.

At 08:00 and 18:00 each day, the experimental dairy goats received feed and were allowed ad libitum access to drinking water and free exercise in the playground. The dairy goat were milked by machine at 17:00 every day. There was no abnormality in rumination, activity, feces and mental state of the dairy goats during the whole process.

### 2.4. Experimental Diet

The experimental diet was formulated according to the nutritional requirements of dairy goats in NRC (2007) [15] and the nutritional requirements of dairy goats in the middle and late lactation periods, with milk production of (1.40 ± 0.50) kg/d and lactating goats at parity 2–3. All diets were isoenergetic and isonitrogenous. CK1 received a cotton straw-free diet, CK2 received a diet containing 20% cotton straw, and YL -11 received a diet containing 20% fermented cotton straw. Table 2 presents the dietary composition and nutrient levels. The experimental diet was provided as a total mixed diet (TMR), with raw materials supplied by the Nala dairy goat farm in Gongliu County, Xinjiang, China.

### 2.5. Sample Collection and Determination

#### 2.5.1. Determination of Safety Performance of Test Strains on Mice

At the end of the experiment, six mice per group were randomly selected, and blood was collected from their retrobulbar venous plexus into heparinized vacuum tubes. Within 30 min after collection, plasma was obtained by centrifugation at 3000×g for 15 min at 4 °C and then immediately used for the following biochemical assays, all of which were completed within 4 h after plasma preparation. Plasma concentrations of interleukin-1β (IL-1β), interleukin-6 (IL-6), and tumor necrosis factor-α (TNF-α) were measured by enzyme-linked immunosorbent assay (ELISA). Colorimetry was applied to determine plasma levels of malondialdehyde (MDA), superoxide dismutase (SOD), glutathione peroxidase (GSH-Px), total antioxidant capacity (T-AOC), and diamine oxidase (DAO). Plasma total protein (TP), total cholesterol (TC), triglyceride (TG), blood urea nitrogen (BUN), glucose (GLU), alanine aminotransferase (ALT), aspartate aminotransferase (AST), and alkaline phosphatase (ALP) were also measured. All assays were performed strictly according to the manufacturer’s instructions supplied with the kits (Beijing Huayin Institute of Biotechnology). After euthanasia, the heart, liver, spleen, lung, and kidney were weighed for calculation of organ indices.

(1) Organ index = organ weight (g)/body weight (g) × 100%.

#### 2.5.2. Production Performance Measurement

After the pre-test period, the initial body weight (IBW) was weighed and the final body weight (FBW) was weighed at the end of the test. The average daily gain (ADG) was calculated: (final body weight—initial body weight)/test days. The feed and residue amounts of the experimental dairy goats were recorded every day, and the daily dry matter intake (DMI) of each dairy goat was calculated.

The dairy goats were driven into the column milking parlor at 17:00 every day and milked once daily using a portable milking machine (Model HT-JN02E, Zhengzhou Hento Machinery Co., Ltd., Zhengzhou, China). The automatic recording dial of the milking machine accurately recorded the daily milk yield of each dairy goat, and the milk yield of each dairy goat was treated as one sample replicate. Average Daily Milk Yield (ADMY) was obtained by dividing the sum of milk yield of each group by the number of dairy goats in the experiment. Feed-to-milk ratio (FMR): average dry matter intake/average milk yield.The 4% fat-corrected milk yield (4% FCMY) [16] was calculated using the following formula:

(2) 4% FCMY = 0.4 × M + 15 × M × F

where M is the average daily milk yield (kg/d) during the experimental period, and F is the measured average milk-fat percentage (%).

#### 2.5.3. Milk Quality Determination

At 17:00 on day 60 of the trial, 50 mL milk samples were collected from both the left and right udder halves of each dairy goat, yielding a total of 100 mL. These samples were immediately transported to the laboratory and analyzed with a multifunctional dairy component analyzer (milkoScan FT3, Foss Analytical Instruments, Denmark, Beijing, China) to determine milk fat percentage (%), milk protein percentage (%), lactose percentage (%), and non-fat milk solids (%).

#### 2.5.4. Determination of Penicillin Content in Milk Samples

Milk samples (50 mL each) were collected from each udder half on day 60 of the trial (left and right) of every dairy goat in the 17:00 group, a total of 100 mL, fully mixed and sub-packed. The milk samples were frozen without preservatives for 15 min in liquid nitrogen and then transferred to a −80 °C freezer for further storage. Penicillin G residue was determined according to “Determination of amoxicillin, Ampicillin, piperacillin, penicillin G, penicillin V, oxacillin, cloxacillin, nafcillin and dicloxacillin residues in milk and milk powder by liquid chromatography-tandem mass spectrometry” [17]; the determination of patulin was according to the “National Standard for Food Safety Determination of patulin in Food” [18]. The detection instrument was a liquid chromatography-tandem mass spectrometer (1290II-6470B, Agilent Technologies Co., LTD., Beijing, China). The sample preparation and detection work were entrusted to Hangzhou Yanqu Information Technology Co., LTD., (Hangzhou, China).

#### 2.5.5. Apparent Nutrient Digestibility

Following completion of the trial period, goat feces samples were collected using the fecal collection method over 4 consecutive days. A total of 16 samples were obtained, 4 per day at 6-h intervals. Each time, 10 g fecal samples were collected rectally with PE gloves, each 100 g fecal sample received 5 mL of 10% sulfuric acid, and mixed evenly. The parallel samples from each dairy goat at different time points were mixed separately and stored at −20 ℃ until tested. Dry matter (DM),Crude protein and crude fat contents in the diet and fecal samples were determined following AOAC methods [19] method, and neutral detergent fiber and acid detergent fiber were measured according to van Soest [20] method.

Nutrient apparent digestibility (%) = 100 − 100 × [nutrient content in the feces (%) × acid insoluble ash content in the feed] / [nutrient content in the diet (%) × acid insoluble ash content in the feces (%)].

#### 2.5.6. Measurement of Serum Indicators

On day 60 of the trial period, a 5-mL blood sample was obtained via jugular vein puncture prior to morning feeding. The samples were subsequently centrifuged at 3000 r/min for 10 min at 4 °C; the upper layer of serum was then taken with a pipettor and transferred into a 1.5-mL Eppendorf tubes, which were frozen in a −20 °C refrigerator. Total protein (TP), albumin (ALB), globulin (GLB), triglyceride (TG), total cholesterol (TC), blood urea nitrogen (BUN), glucose (GLU), alanine aminotransferase (ALT), aspartate aminotransferase (AST) and serum alkaline phosphatase (ALP) were measured by a Mindray BS-420 automatic biochemical instrument. Serum superoxide dismutase (SOD), glutathione peroxidase activity (GSH-Px), total antioxidant capacity (T-AOC), catalase (CAT) and malondialdehyde (MDA) were measured by colorimetry using an enzyme-labeled analyzer (DR-200BS, Wuxi Huawei Instrument Co., LTD., Jiangsu, China). The levels of tumor necrosis factor-α (TNF-α), interleukin-1β (IL-1β), transforming growth factor-β1 (TGF-β1) and interleukin-10 (IL-10) in the serum were detected by enzyme-linked immunosorbent assay (ELISA). The above indicators were detected using the kit of the Beijing Huaying Institute of Biotechnology, and the operation instructions were strictly followed.

#### 2.5.7. Rumen Fluid Sample Collection and Determination

Six goats from each group were randomly selected on day 60 of the trial period. Following 12 h of feed withdrawal and 2 h of water deprivation, rumen fluid was obtained via oral gavage with a stomach tube at approximately 12 h after the last feeding (08:00). The initial 50 mL of rumen fluid was removed to avoid saliva contamination, and then approximately 30 mL of rumen fluid was collected. Immediately after collection, the pH value was determined using a portable pH meter (Shanghai INESA Scientific Instrument Co., LTD., Shanghai, China), and the average value was taken from three consecutive readings. The remaining rumen fluid was aliquoted into 15 mL and 5 mL cryogenic tubes and stored at −20 °C and −80 °C, respectively, for subsequent analysis of rumen fermentation parameters and microbial community. The phenol -sodium hypochlorite colorimetric method [21] was employed to measure ammonia nitrogen concentration, while gas chromatography was used to quantify volatile fatty acid concentrations [22].

#### 2.5.8. DNA Extraction, PCR Amplification and Sequencing Analysis of Ruminal Bacteria

Total genomic DNA was extracted from rumen fluid samples using a DNA extraction kit (DM401-C3-P2, Nanjing Novogene Biotechnology Co., Ltd., Nanjing, China) in accordance with the manufacturer’s instructions. An Agilent 5400 Fragment Analyzer (Agilent Technologies, Beijing, China) was used to assess DNA integrity, A Qubit fluorometer (Thermo Fisher Scientific, Waltham, MA, USA) and a NanoDrop spectrophotometer (Thermo Fisher Scientific, Wilmington, DE, USA) were used to determine DNA concentration and purity, respectively.

For PCR amplification, the bacterial 16S rRNA gene target region was amplified using universal bacterial primers 27F (5′-AGRGTTTGATYNTGGCTCAG-3′) and 1492R (5′-TASGGHTACCTTGTTASGACTT-3′). Purified and quantified PCR products were pooled for library construction. After quality evaluation, The qualified libraries were sequenced on an Illumina MiSeq PE300 platform (Illumina, San Diego, CA, USA). Subsequently, the raw reads were subjected to quality filtering and trimming, after which paired-end reads were assembled into contigs. Raw sequencing data were submitted to the Sequence Read Archive (SRA) of the National Center for Biotechnology Information (NCBI) and are available under BioProject accession PRJNA1453688.

Bioinformatic analyses were performed within the QIIME2 pipeline. The DADA2 algorithm was applied for sequence denoising to produce amplicon sequence variants (ASVs). Representative ASV sequences were taxonomically classified using the QIIME2 feature-classifier plugin with the SILVA reference database. The ANCOM method was applied to detect differentially abundant taxa among groups. Alpha- and beta-diversity (PCoA) were calculated using QIIME2 core-diversity metrics to compare microbial richness and community structure among groups.

### 2.6. Data Statistics and Analysis

Experimental data were first organized using Microsoft Excel 2016. Statistical analyses were performed with SPSS 24.0 software (IBM Corp., Armonk, NY, USA). Means among the three groups were compared by one-way analysis of variance (ANOVA) followed by Duncan's multiple range test. Data are presented as mean ± standard deviation (SD). Differences were regarded as significant at *p* < 0.05 and highly significant at *p* < 0.01. In this study, Pearson correlation analysis was used to evaluate the relationships among average daily milk yield (ADMY), 4% fat-corrected milk (4% FCMY), feed-to-milk ratio, and rumen microbiota. Additionally, the Mantel test was performed using the SPLS Life Science Statistical Platform (https://spls.cn/#/, accessed on 15 October 2025) to assess the overall association between rumen microbiota and the above-mentioned milk-production-related traits, and a corresponding correlation heatmap was generated.

## 3. Results

### 3.1. Safety Evaluation of Strain YL-11

#### 3.1.1. Body Weight and Organ Index of Mice

As shown in Table 3, there was no significant difference in the initial body weight of mice between the control and YL-11 groups (*p* > 0.05). A significantly higher final body weight was observed in the YL-11 group than in the control group (*p* > 0.05). Heart index, liver index, spleen index, lung index, and kidney index did not differ significantly between the two groups (*p* > 0.05).

#### 3.1.2. Blood Index Results of Mice

Table 4 summarizes the serum biochemical and immune parameters. Between the two groups, no significant differences were found in total protein (TP), total cholesterol (TC), triglyceride (TG), blood urea nitrogen (BUN), aspartate aminotransferase (AST), alanine aminotransferase (ALT), or alkaline phosphatase (ALP) (*p* > 0.05). Blood glucose (GLU) also showed no significant difference between the YL-11 and control groups (*p* > 0.05). Furthermore, YL-11 administration had no significant effect on tumor necrosis factor-α (TNF-α), interleukin-1β (IL-1β), interleukin-6 (IL-6), or diamine oxidase (DAO), and no significant differences were observed in superoxide dismutase (SOD), malondialdehyde (MDA), or total antioxidant capacity (T-AOC) in mice (*p* > 0.05).

### 3.2. Influence of YL-11 Fermented Cotton Straw on the Performance of Dairy Goats

As shown in Table 5, no significant effect of different treatment groups on the IBW was observed, FBW, ADG and DMI of dairy goats (*p* > 0.05). The yields of ADMY and 4% FCMY in YL-11 group were markedly higher than those in the CK1 and CK2 groups (*p* < 0.01). Accordingly, the YL-11 group exhibited a significantly lower FMR than the CK2 group (*p* < 0.01).

### 3.3. Influence of YL-11 Fermented Cotton Straw on Apparent Nutrient Digestibility of Dairy Goats

Table 6 shows that DM apparent digestibility was significantly greater in the CK1 group than in the CK2 and YL-11 groups (*p* < 0.05). The apparent digestibility of CP and Ash did not differ significantly between the CK1 and YL-11 groups (*p* > 0.05), but both were significantly higher than those in the CK2 group (*p* < 0.01). NDF apparent digestibility in the CK1 group was significantly higher than that in the CK2 group (*p* < 0.01). For ADF, no significant effect was observed among the different treatment groups of dairy goats (*p* > 0.05).

### 3.4. Influence of YL-11 Fermented Cotton Straw on Milk Quality of Dairy Goats

As presented in Table 7, the YL -11 group showed a significantly higher milk fat content than the CK1 and CK2 groups (*p* < 0.01), as well as a significantly higher lactose rate than the CK1 and CK2 groups (*p* < 0.05). Different treatment groups did not significantly affect the milk protein percentage or non-fat milk solid percentage of dairy goats ( *p* > 0.05).

### 3.5. Influence of YL-11 Fermented Cotton Straw on Penicillin Content in Milk from Dairy Goats

Table 8 indicates that patulin and penicillin G concentrations in milk samples from dairy goats in the CK1, CK2, and YL-11 groups were below the method detection limit (BLQ), and neither target compound was detected in any group.

### 3.6. Influence of YL-11 Fermented Cotton Straw on Antioxidant and Immune Properties of Dairy Goats

Table 9 reveals that T-AOC, SOD, CAT, and GSH-Px were significantly higher in the YL-11 group than in the CK1 and CK2 groups (*p* < 0.01). The YL -11 group showed significantly lower MDA and TNF-α levels than the CK2 group (*p* < 0.05). The YL -11 group had significantly lower IL-1β and TGF-β1 levels than the CK1 and CK2 groups (*p* < 0.01). IL-10 level was significantly higher in the YL-11 group than in the CK1 group (*p* < 0.01).

### 3.7. Influence of YL-11 Fermented Cotton Straw on Blood Biochemical Parameters of Dairy Goats

Table 10 reveals that the YL -11 group had significantly higher TP and GLB than the CK2 group (*p* < 0.01). ALB was significantly lower in the YL-11 group than in the CK1 and CK2 groups (*p* < 0.01). Similarly, ALP was significantly lower in the YL-11 group than in the CK1 group (*p* < 0.05). No significant effects on TC, TG, BUN, GLU, AST, or ALT were observed among the different treatment groups (*p* > 0.05).

### 3.8. Influenc of YL-11 Fermented Cotton Straw on Rumen Fermentation in Dairy Goats

Table 11 demonstrates that the CK2 group had a significantly higher pH than the CK1 and YL-11 groups (*p* < 0.01). In the YL-11 group, NH3-N and the acetic acid/propionic acid ratio were significantly higher than those in the CK1 and CK2 groups (*p* < 0.05). The CK1 group showed significantly higher Valeric acid than the CK2 group (*p* < 0.05). Among the different treatment groups of dairy goats, no significant effects were found on acetic acid, propionic acid, butyric acid, isobutyric acid, isovaleric acid, or TVFA ( *p* > 05).

### 3.9. Influence of YL-11 Fermented Cotton Straw on Rumen Microbial Diversity in Dairy Goats

Thirty-nine OTUs were unique to the CK1 group. Forty-eight OTUs were unique to the CK2 group. Thirty-five OTUs were unique to the YL-11 group (Figure 1a). In PCoA analysis, PC1 and PC3 explained 15.42% and 7.87% of the total variance, respectively (Figure 1b). PCoA and PLS-DA analyses indicated distinct clustering patterns among the three groups, although some overlap was present between groups, suggesting that YL-11 fermentation modulated the rumen microbial community structure (Figure 1b,c). The PLS-DA results (Figure 1c) were consistent with the PCoA analysis, showing clear separation between the CK2 and YL-11 groups.

Figure 2 presents the microbial composition differences among the groups. At the phylum level, Verrucomicrobiota abundance was significantly greater in the YL-11 group than in the CK1 and CK2 groups (*p *< 0.05), whereas Bacteroidota abundance was higher in the YL-11 group but did not differ significantly from the CK1 and CK2 groups (Figure 2a). At the genus level, uncultured_rumen_bacterium was significantly enriched in the YL-11 group compared with the CK1 and CK2 groups (*p* < 0.05), while Prevotella showed a higher abundance in the YL-11 group without a significant difference relative to the CK1 and CK2 groups (Figure 2b).

LEfSe analysis was conducted to further identify the species showing significant differences between the groups. As shown in Figure 2c, the transverse columnar shape represents a species, and the length represents the LDA score, and the higher the score, the more significant the difference is. The CK2 group is missing in the figure, indicating that there is no species with significant difference in this group. When LDA = 4, in the CK1 group, *c_Clostridia*, *f__unclassified_Bacteria*, *g__unclassified_Bacteria*, *c__unclassified_Bacteria*, *p__unclassified_Bacteria*, *g___Ruminococcus__gauvreauii_group*, and *g__unclassified_Lachnospiraceae* were seven potential microbial markers. In the YL-11 group, *p_Verrucomicrobiota*, *c_Kiritimatiellae*, *o_WCHB1-41*, *f_uncultured_rumen_bacterium*, *f_uncultured_rumen_bacterium* and *g_uncultured_rumen_bacterium* were five potential microbial markers.

As shown in Figure 3, when the Mantel test and Pearson correlation coefficient were used to analyze the correlation between lactation performance and dominant bacteria, ADMY and *Prevotella*, uncultured rumen bacterium, *Rikenellaceae_RC9_gut_group*, *NK4A214_group*, and Verrucomicrobia were positively correlated. The 4% FCMY was positively correlated with *Prevotella*, uncultured rumen bacterium, *Rikenellaceae_RC9_gut_group*, *NK4A214_group*, Verrucomicrobia, etc. The FMR and *Prevotella*, uncultured rumen bacterium, *Rikenellaceae_RC9_gut_group*, *NK4A214_group*, Verrucomicrobia, *Cyanobacteria*, and *Synergistetes* were positively correlated. The ADMY, 4% FCMY, and FMR were all negatively correlated with *Firmicutes*, *Desulfovibrio*, *Fibrobacterota*, *Patescibacteria*, etc.

## 4. Discussion

### 4.1. In Vivo Safety Evaluation of Penicillium chrysogenum YL-11

The biosafety of microbial strains is a core prerequisite for their development and application in feed additives and fermentation inoculants [23]. This study systematically evaluated the in vivo safety of *Penicillium chrysogenum* YL-11 (CCTCC M 20261726) in mice across growth performance, organ development, serum biochemistry, oxidative stress and inflammatory status, to offer a theoretical basis for its application in cotton straw fermentation and ruminant feed production.

Organ index is a fundamental indicator for assessing target organ injury induced by exogenous substances [24]. Oral gavage of YL-11 caused no significant differences in the organ indices of heart, liver, spleen, lung and kidney (*p* > 0.05), indicating that the strain did not induce visceral pathological changes at the tested dose. Meanwhile, the final body weight of mice in the YL-11 group was significantly higher than that in the control group (*p* < 0.05). The significantly higher final body weight of mice in the YL-11 group may be attributed to multiple factors, including potential improvements in feed intake, intestinal development, or metabolic efficiency, rather than solely to a direct growth-promoting effect of the strain.

Serum biochemical parameters reflect hepatic and renal function as well as glucose and lipid homeostasis, and serve as core markers for evaluating test-substance-induced hepatotoxicity and nephrotoxicity [25]. AST, ALT and ALP are classic biomarkers of hepatocellular injury; damage to hepatocyte membranes by exogenous compounds triggers massive release of intracellular transaminases into the bloodstream [26]. In the present study, serum AST, ALT, ALP, BUN, TC, and TG did not differ significantly between the two groups (*p* > 0.05), suggesting that *P. chrysogenum* YL-11 caused no hepatic or renal impairment, nor did it disrupt basal lipid metabolism in mice. Previous studies have confirmed that certain *Penicillium* species lower blood glucose via regulating intestinal carbohydrate degradation and improving insulin sensitivity. This represents a metabolic regulatory effect of the strain, rather than abnormal blood glucose caused by toxic injury, and the underlying hypoglycemic mechanism warrants further investigation.

Intestinal antioxidant capacity, barrier integrity and inflammatory cytokine levels reflect the impact of microbial strains on intestinal homeostasis. YL-11 administration caused no significant changes in antioxidant indices (SOD, T-AOC, MDA), the intestinal barrier marker DAO, or the pro-inflammatory cytokines (TNF-α, IL-1β and IL-6 (*p* > 0.05).

### 4.2. Influence of YL-11 Fermented Cotton Straw on Performance, Apparent Digestibility and Milk Quality of Dairy Goats

Growth performance and feed intake are core indicators for evaluating the feeding value of roughage resources. Fermented feed has been widely reported to improve the lactation performance and health status of ruminants via enhancing diet palatability, promoting nutrient digestion and modulating rumen function [27,28]. In the present study, initial body weight and dry matter intake did not differ significantly across all groups of dairy goats (*p* > 0.05), which confirmed the uniform body condition of experimental animals and eliminated the interference of baseline differences in subsequent results. Meanwhile, it indicated that *Penicillium chrysogenum* YL-11 (CCTCC M 20261726) fermented cotton straw exerted no adverse effect on the feed intake of dairy goats, and could be used as a conventional roughage source in diets. The YL-11 group showed significantly higher average daily milk yield (ADMY) and 4% fat-corrected milk yield (4% FCMY). Moreover, a significantly lower feed-to-milk ratio was observed compared with the CK1 and CK2 groups (*p* < 0.01). This finding was in agreement with the results of Li et al. [29], who reported that dietary inclusion of fermented feed at 10% proportion significantly increased ADMY and 4% FCMY in Holstein cows without affecting feed intake. Collectively, fungal fermentation is an effective strategy to upgrade low-quality straw resources and improve ruminant production performance.

Nutrient apparent digestibility is a core parameter for feed quality evaluation, diet formulation optimization and production efficiency improvement. In this study, in the YL-11 group, the apparent digestibility of dry matter (DM), ether extract (EE), neutral detergent fiber (NDF), and acid detergent fiber (ADF) was numerically greater than that in the CK2 group, although the difference was not statistically significant (*p* > 0.05). The apparent digestibility of crude protein (CP) and crude ash was significantly higher in the YL-11 group than in the CK2 group (*p* < 0.05), and the magnitude of improvement was in line with previous reports [30]. Zheng et al. [30] demonstrated that treatment of rapeseed straw with lignin-degrading fungi Ceriporiopsis subvermispora and Lentinula edodes increased in vitro gas production by 86% and 20%, respectively, confirming that fungal lignin degradation effectively improved the feeding value of straw. The improvement of CP digestibility can be mainly attributed to two aspects. First, microbial proliferation during solid-state fermentation leads to the synthesis of bacterial protein, which increases the content of available nitrogen sources in the diet. Second, partial degradation of lignin breaks the cross-link structure between lignin and protein, releasing the protein originally encapsulated in the lignocellulose matrix and improving its accessibility to rumen microorganisms [31]. Yu et al. [32] found that Pleurotus ostreatus fermentation increased the crude protein content of distillers’ grain waste to approximately 130 g/kg DM and decreased the lignin content to 5%, which further verified the close correlation between lignin degradation and protein release. The simultaneous significant increase in crude ash digestibility in the YL-11 group also corroborated this release effect: after partial lignin degradation, the bound mineral elements were released and more efficiently absorbed and utilized by animals [33].

Milk composition is a core determinant of milk nutritional quality. Previous studies have confirmed that fermented feed supplementation can modulate milk composition; for example, dietary fermented soybean meal or *Saccharomyces cerevisiae* culture significantly increased milk fat percentage in Holstein cows [34,35,36,37]. In the present study, dietary inclusion of YL-11 fermented cotton straw resulted in significantly higher milk fat percentage (*p* < 0.01) and lactose percentage (*p* < 0.05) than in the CK1 and CK2 groups, suggesting that fermented straw modulates lactation performance of dairy goats via multiple pathways. The underlying mechanisms mainly include two aspects. First, probiotic components from YL-11 fermented cotton straw contribute to optimizing the rumen microbial ecosystem, improving rumen fermentation efficiency and promoting nutrient degradation and absorption [38,39]. Second, bioactive components produced during fermentation may enhance milk yield and promote milk fatty acid synthesis by regulating metabolic pathways in mammary gland tissues.

### 4.3. Influence of YL-11 Fermented Cotton Straw on Penicillin Content in Milk of Dairy Goats

Penicillin can trigger intestinal flora disturbance and allergic reactions in animals and fails to satisfy safety thresholds for fresh milk [40]. As a typical secondary metabolite of *Penicillium* fungi, patulin possesses cytotoxic and immunotoxic properties and may accumulate in milk via lactation. Accordingly, this study simultaneously determined the concentrations of penicillin G and patulin in milk samples [41]. In the present study, the concentrations of penicillin G and patulin in milk from both control groups and the YL-11 fermented-cotton-straw treatment group were below the method-defined quantitative detection limit. These findings demonstrated that feeding YL-11 fermented cotton straw would not result in penicillin or mycotoxin residues in fresh goat milk. Although *Penicillium chrysogenum* serves as the classic industrial strain for penicillin manufacturing, its penicillin biosynthesis heavily relies on submerged liquid-fermentation systems and specific side-chain precursors [42]. In this experiment, cotton straw solid-state fermentation took lignocellulose as primary substrate. Lacking the metabolic microenvironment and precursor substances required for penicillin biosynthesis, YL-11 could not synthesize or accumulate penicillin under such fermentation conditions. The absence of patulin in milk further validated the biosafety of strain YL-11, which is in good accordance with the above-mentioned mouse in vivo toxicological outcomes. This result supplies critical food safety evidence supporting feed development and the industrial application of strain YL-11.

### 4.4. Influence of YL-11 Fermented Cotton Straw on Blood Antioxidant, Immune Properties and Blood Biochemical Indexes of Dairy Goats

The antioxidant system is tightly associated with immune function in ruminants, as enhanced antioxidant capacity alleviates inflammatory responses and improves immune performance [43]. In the present study, compared with the CK1 and CK2 groups, the YL-11 group showed a significantly higher T-AOC level, significantly higher SOD, CAT, and GSH-Px activities, and lower MDA, TNF-α, IL-1β, and TGF-β1 contents (*p* < 0.05), suggesting that YL-11 fermented cotton straw enhanced both antioxidant capacity and immune function in dairy goats.

This beneficial effect may be driven by two pathways. First, YL-11 fermentation degrades anti-nutritional factors and toxic components in raw cotton straw, thus reducing oxidative stress caused by exogenous substances [44]. Second, microbial metabolism during fermentation generates bioactive components such as antioxidant peptides and polyphenols, which modulate immune cytokine secretion and exert synergistic antioxidant and immunomodulatory effects [45]. The significantly elevated serum GLB level in the YL-11 group further corroborated the improved immune function, as GLB is mainly composed of immunoglobulins and reflects antibody-mediated immune response intensity.

For serum biochemical indices, no significant differences were found in TC, TG, GLU, ALT and AST among all groups (*p* > 0.05), confirming that YL-11 fermented cotton straw caused no adverse effects on hepatic and renal function or glucose and lipid homeostasis. The YL-11 group had significantly higher TP and GLB contents and lower ALB content than the control groups (*p* < 0.05), suggesting that fermented cotton straw primarily modulated protein metabolism in dairy goats. In the YL-11 group, serum BUN exhibited a numerical decrease, although the difference did not reach statistical significance, suggesting a tendency toward improved nitrogen utilization that warrants further investigation [46].

### 4.5. Influence of YL-11 Fermented Cotton Straw on Rumen Fermentation Parameters of Dairy Goats

Rumen fermentation parameters serve as key indicators of rumen microenvironment stability and fermentation efficiency, and alterations in these parameters directly influence nutrient digestion and absorption in ruminants [47]. Rumen pH reflects the overall fermentation status, and its normal range in dairy goats is 5.5–7.5 [48]. Beyond this range, microbial activity is suppressed, thereby impairing fiber degradation and nutrient metabolism. In the present experiment, rumen pH varied from 6.10 to 6.28 among all groups, suggesting that YL-11 fermented cotton straw had no adverse effect on rumen fermentation.

Ammonia-nitrogen (NH3-N) functions as the principal nitrogen source for ruminal microbial protein synthesis, and its optimal concentration is 0.35–33.92 mg/dL [49,50,51,52]. In the present study, NH_3_-N concentrations ranged from 13.18 to 16.75 mg/dL across the groups, with all values remaining within the normal range. The YL-11 group showed significantly higher NH_3_-N than the CK1 and CK2 groups (*p* < 0.05), but it still remained within the suitable range, potentially indicating improved protein degradation efficiency without adverse effects on microbial metabolism.

Volatile fatty acids (VFAs) are the primary products of carbohydrate fermentation and serve as the main energy source for dairy goats, with their composition affecting energy utilization and milk fat synthesis [53]. In the present study, the acetate/propionate ratio was significantly higher in the YL-11 group than in the other groups (*p* < 0.05), whereas total VFA concentration exhibited a numerical increase without significant differences among groups (*p* > 0.05). This may be attributed to enhanced microbial diversity and a greater relative abundance of fiber-degrading bacteria, which promoted cellulose degradation and VFA production. The elevated acetate/propionate ratio in the YL-11 group, relative to CK1 and CK2, indicates a shift toward acetate-type fermentation, thereby supplying more energy for the goats.

### 4.6. Influence of YL-11 Fermented Cotton Straw on Rumen Microbial Diversity of Dairy Goats

Probiotics and their metabolites in fermented feed can modulate the rumen microbial community, increase microbial richness and diversity, and produce organic acids that suppress pathogenic bacteria, thereby promoting beneficial bacteria and maintaining rumen ecological balance [54]. In this study, the YL-11 group had 35 unique OTUs, while the CK1 and CK2 groups had 39 and 48 unique OTUs, respectively. Despite a numerically lower number of unique OTUs in the YL-11 group, YL-11 fermentation markedly altered the overall microbial diversity and community structure, as revealed by PCoA and PLS-DA analyses. Indicating that YL-11 treatment induced a substantial restructuring of the rumen microbial community. This may result from changes in substrate availability, metabolite profiles, or rumen pH, which affect microbial colonization and competition [55]. At the phylum and genus levels, the YL-11 group exhibited a significantly higher relative abundance of Verrucomicrobiota, whereas Bacteroidota and Prevotella displayed increasing trends that did not reach statistical significance. Verrucomicrobiota has been documented to participate in plant polysaccharide degradation [56], and its increased abundance may reflect enhanced accessibility of fibrous substrates in YL-11 fermentation products, promoting the proliferation of related microorganisms. LEfSe analysis identified five potential biomarkers in the YL-11 group, including *p_Verrucomicrobiota* and *c_Kiritimatiellae*, which participate in fiber degradation and short-chain fatty acid synthesis [57,58]. Additionally, the genus*uncultured_rumen_bacterium* was significantly enriched in the YL-11 group, suggesting a potentially important role that warrants further isolation and identification. Close associations between rumen microbes and lactation performance were revealed by Mantel test and Pearson correlation analysis. The abundance of *Prevotella*, an important carbohydrate-degrading genus, was positively correlated with feed-to-milk ratio and milk yield, consistent with its role in fiber decomposition and energy metabolism [59,60]. Likewise, the positive correlation between *Verrucomicrobiota* and lactation performance further supports its potential contribution to host nutrient utilization efficiency.

## 5. Conclusions

The current study was conducted to assess the biosafety of *Penicillium chrysogenum* YL-11 (CCTCC-M-20261726) and verify its feeding effects on lactation performance, milk quality, ruminal fermentation, and rumen microbiota in dairy goats. The mouse safety assessment confirmed that YL-11 caused no adverse changes in organ indices, serum biochemistry, or immune function, demonstrating reliable in vivo biosafety. In dairy goats, dietary supplementation with YL-11-fermented cotton straw optimized ruminal fermentation profiles, enriched fiber-degrading microbiota, and enhanced systemic antioxidant and immune capacities, which collectively improved lactation performance and milk quality and reduced the feed-to-milk ratio. In conclusion, YL-11 fermentation is a safe and effective strategy to enhance the feeding value of cotton straw and exhibits great application potential in dairy goat production. Further large-scale and long-term feeding trials are still needed to validate these beneficial effects and promote the practical application of this microbial fermentation technology in ruminant feeding.

## Figures and Tables

**Figure 1:**
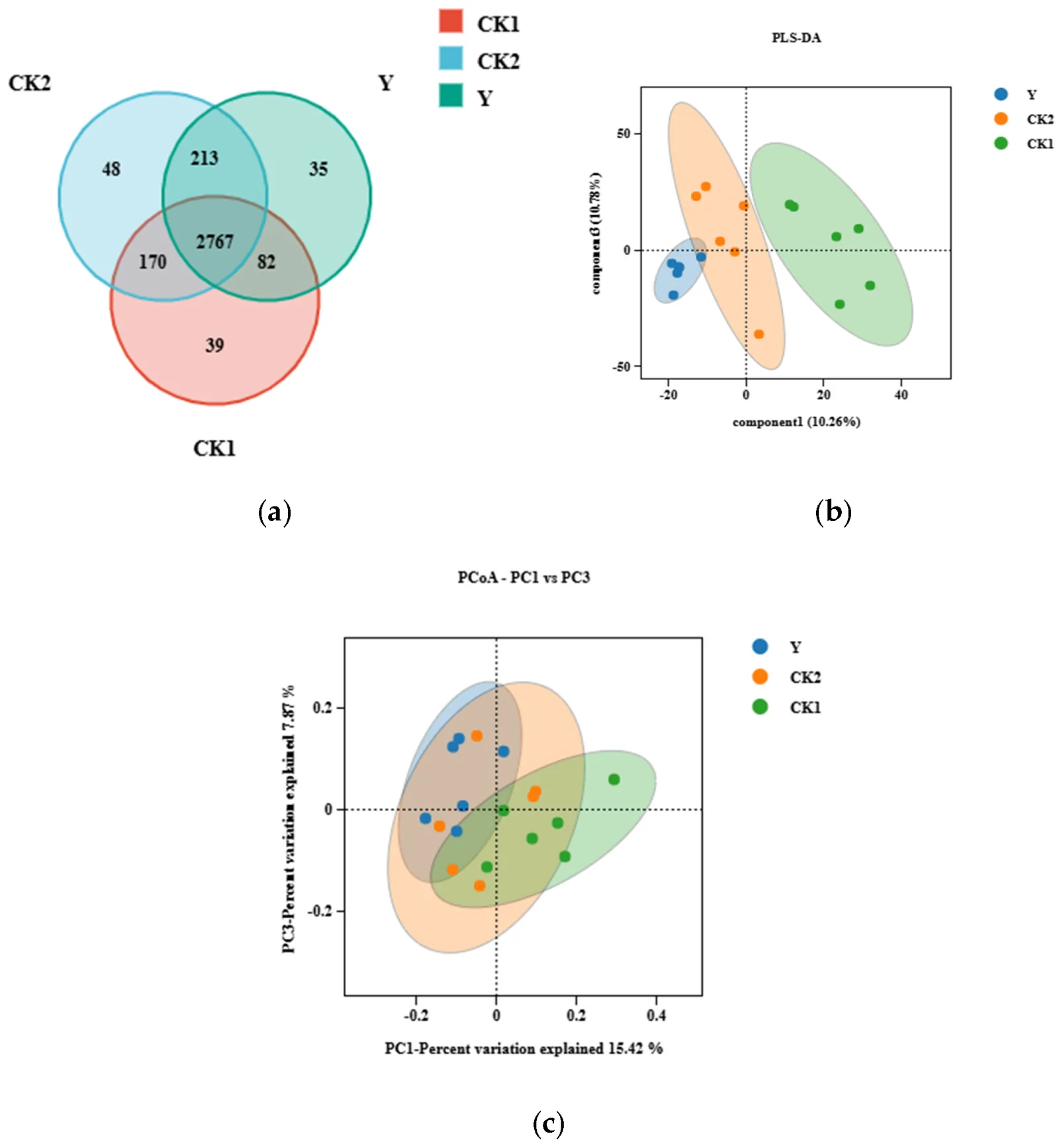
Rumen microbial community analysis among the three groups (CK1, CK2, and YL-11). (**a**) Venn diagram displaying shared and unique OTUs among the three groups. (**b**) Principal coordinate analysis (PCoA) plot generated from weighted UniFrac distances. (**c**) Partial least squares discriminant analysis (PLS-DA) plot illustrating distinct clustering of microbial communities among the three groups. CK1, green; CK2, orange; YL-11, blue.

**Figure 2:**
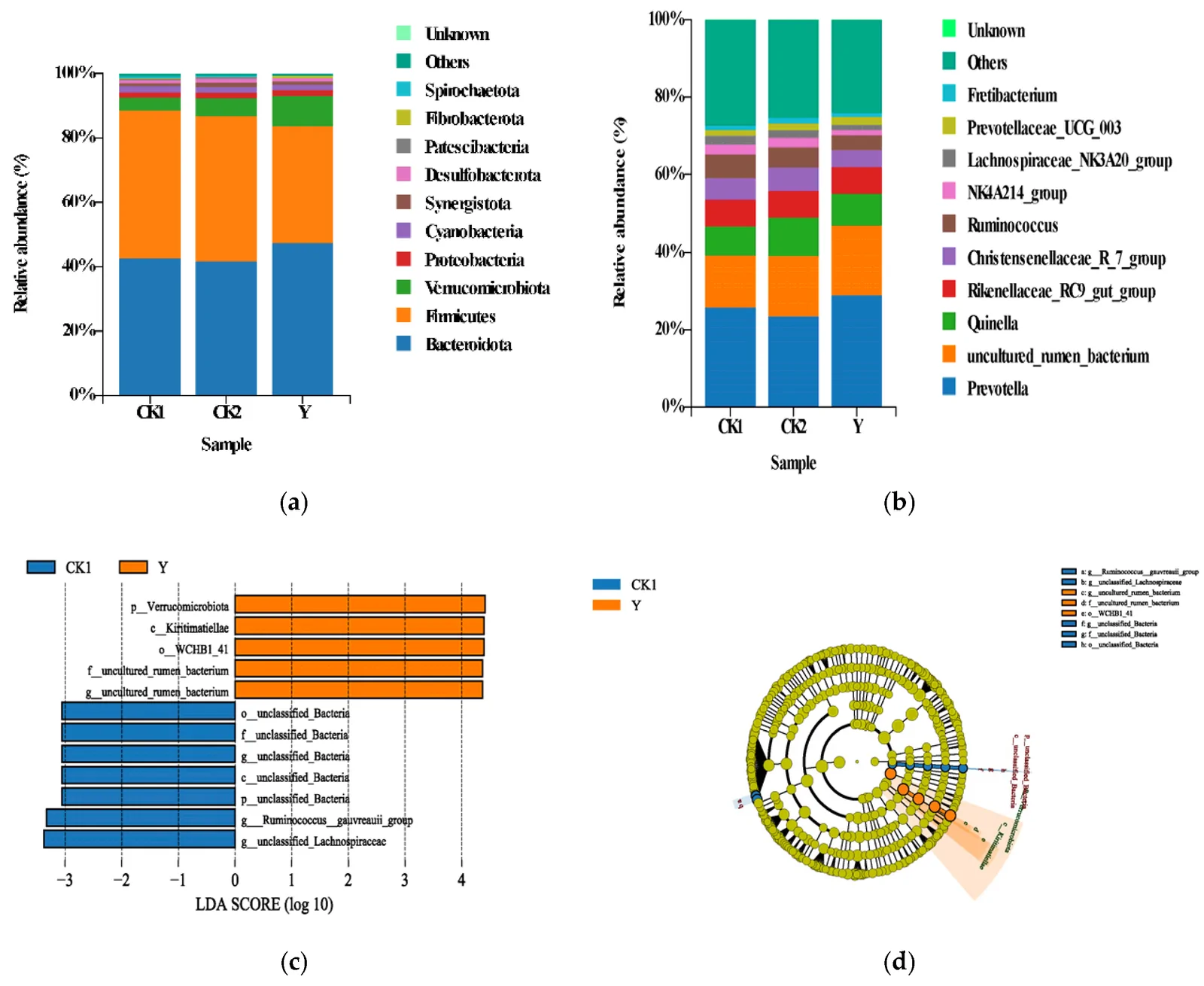
(**a**) Bacterial community composition at the phylum level; (**b**) bacterial community composition at the genus level; (**c**) LDA score bar plot of LEfSe analysis, blue represents the CK group, and orange represents the YL-11 group; (**d**) LEfSe cladogram.

**Figure 3:**
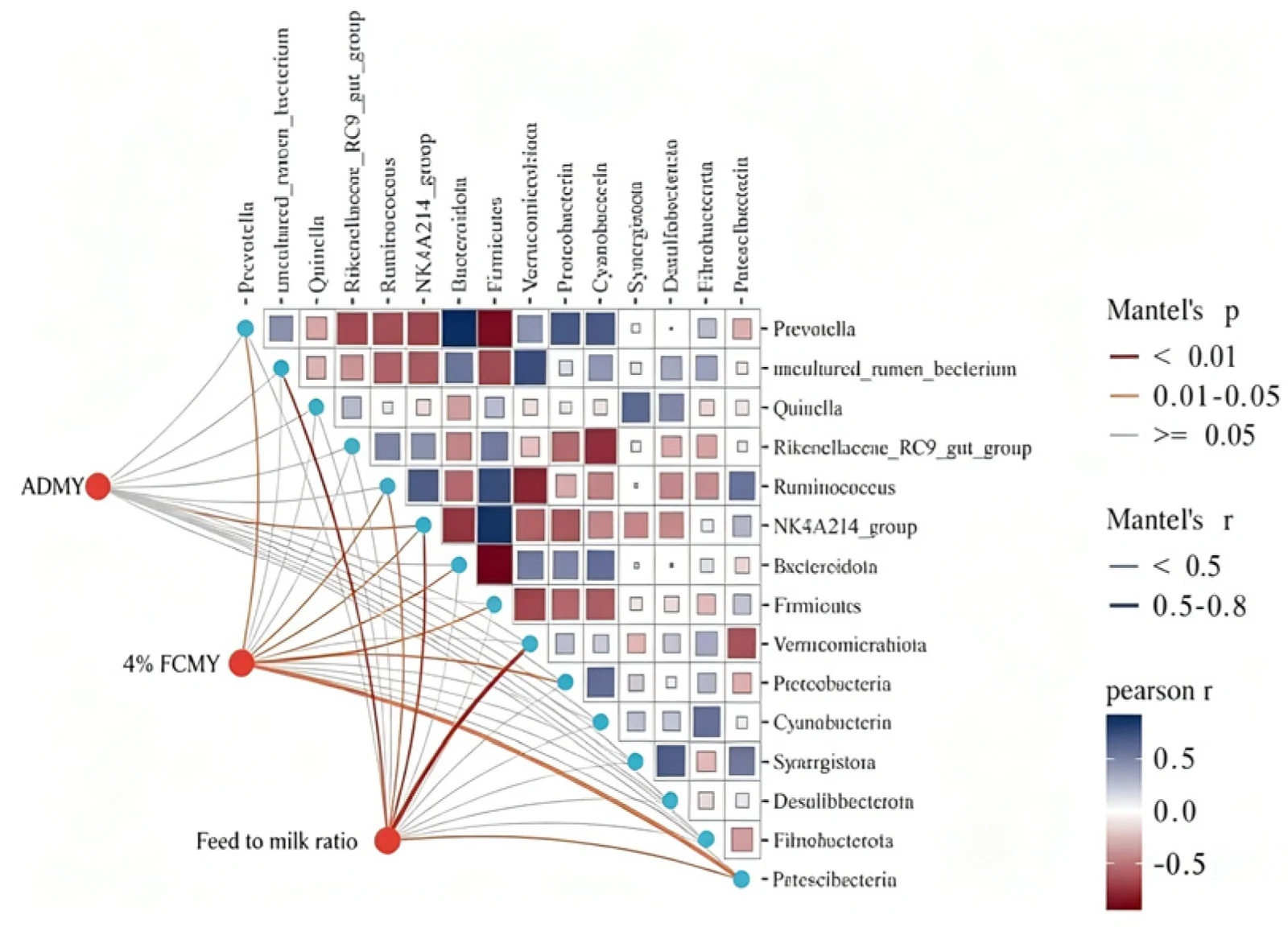
Association analysis between lactation performance and dominant bacterial flora.

**Table 1:** Nutrient levels of cotton straw and fermented cotton straw (dry matter basis, %).

Items	Cotton Straw	Fermented Cotton Straw
Dry matter DM	94.00	35.00
Metabolizable energy ME ^1^ (MJ/kg)	9.21	9.58
Crude protein CP	9.74	10.01
Neutral detergent fiber NDF	44.83	41.55
Acid detergent fiber ADF	35.9	32.99
Calcium Ca	0.38	0.38
Phosphorus P	0.11	0.11
Free gossypol FG (mg/kg)	2.00	1.00
Trimethoprim (μg/kg)	- ^3^	BLQ ^2^
*Patulin* (μg/kg)	-	BLQ
*Penicillin* G (μg/kg)	-	BLQ

^1^ ME was calculated based on NRC (2007) [15] , the rest are measured values. Table 2 is the same. ^2^ BLQ indicates that the detection value is below the lower limit of quantification or the target analyte is not detected in the sample. ^3^ “-” indicates not detected.

**Table 2:** Ingredients and nutrient levels for the experimental diet (dry matter basis, %).

Items	CK 1	CK 2	YL-11
Raw Ingredients			
Corn silage	13.63	4.90	2.50
Wheat straw	11.76	12.80	15.02
Alfalfa	29.43	17.32	17.50
Cotton straw	0.00	20.00	20.00
Wheat bran	4.64	4.80	4.80
Steam-flaked corn	17.06	16.25	15.50
Concentrate supplement ^1^	21.58	22.13	22.88
Calcium hydrogen phosphate CaHPO_4_	1.90	1.80	1.80
Total	100	100	100
nutritional level ^2^			
Metabolizable energy ME (MJ/kg)	9.81	9.81	9.81
Crude protein CP	10.95	10.95	10.95
Neutral detergent fiber NDF	42.19	39.42	38.74
Acid detergent fiber ADF	23.62	24.57	24.37
Calcium Ca	1.16	1.16	1.16
Phosphorus P	0.64	0.64	0.64

^1^ Concentrate supplement was purchased from Tiankang (Xinjiang) Feed Co., Ltd., (Wujiaqu, China) containing corn, soybean meal, cotton meal, bran, corn husk, calcium hydrogen phosphate, stone powder, sodium chloride (NaCl), amino acids, trace elements, and vitamins. Its crude protein content ≥ 18.0%, crude fiber content ≤ 24.0%, crude ash content ≤ 16.0%, calcium 0.50–1.50%, total phosphorus ≥ 0.40%, sodium chloride 0.30–1.50%, and lysine ≥ 0.30%. ^2^ For the nutritional level, the measured value was used, while ME was calculated.

**Table 3:** Influence of strain YL-11 on organ indices in mice (*n* = 6).

Items	CK	YL-11	*p*-Value
Initial weight (g)	31.67 ± 4.08	31.75 ± 2.08	0.15
Final weight (g)	40.00 ± 2.22	50.10 ± 1.16	<0.05
Cardiac index (%)	0.58 ± 0.22	0.51 ± 0.16	0.559
Hepatic index (%)	4.61 ± 0.53	5.15 ± 0.58	0.121
Spleen index (%)	0.32 ± 0.01	0.33 ± 0.06	0.855
Pulmonary index (%)	0.67 ± 0.09	0.70 ± 0.10	0.689
Renal index (%)	1.43 ± 0.29	1.45 ± 0.24	0.874

Values are presented as mean ± SD. *p* < 0.05 indicates a significant difference between groups.

**Table 4:** Influence of strain YL-11 on serum biochemical, antioxidant, and immune parameters in mice (*n* = 6).

Items	CK	YL-11	*p*-Value
TP (g/L)	55.67 ± 5.94	53.38 ± 3.66	0.440
TC (mmol/L)	1.63 ± 0.40	1.81 ± 0.39	0.484
TG (mmol/L)	0.87 ± 0.41	1.06 ± 0.72	0.093
BUN (mg/dL)	21.55 ± 1.24	21.68 ± 0.93	0.102
GLU (mmol/L)	6.86 ± 0.61	5.87 ± 0.91	0.052
AST (U/L)	248.83 ± 0.43	269.17 ± 0.44	0.444
ALT (U/L)	51.29 ± 7.18	46.28 ± 0.12	0.413
ALP (U/L)	117.94 ± 16.72	109.38 ± 10.90	0.325
TNFα (pg/mg.prot)	4.76 ± 0.27	5.53 ± 0.22	0.113
IL-1β (pg/mg.prot)	1.64 ± 0.10	1.96 ± 0.18	0.237
IL-6 (pg/mg.prot)	7.85 ± 0.43	7.98 ± 0.42	0.307
DAO (U/mg.prot)	1.07 ± 0.23	1.45 ± 0.28	0.210
SOD (U/mg.prot)	2.45 ± 1.55	2.99 ± 1.20	0.510
MDA (nmol/mg.prot)	0.81 ± 2.15	1.43 ± 0.25	0.060
T-AOC (U/mg.prot)	4.38 ± 0.15	4.83 ± 0.60	0.140

Data are expressed as mean ± SD. The abbreviations are defined as follows: TP for total protein; TC for total cholesterol; TG for triglyceride; BUN for blood urea nitrogen; GLU for glucose; AST for aspartate aminotransferase; ALT for alanine aminotransferase; ALP for alkaline phosphatase; TNF-α for tumor necrosis factor-α; IL-1β for interleukin-1β; IL-6 for interleukin-6; DAO for diamine oxidase; SOD for superoxide dismutase; MDA for malondialdehyde; T-AOC for total antioxidant capacity.

**Table 5:** Influence of YL-11 fermented cotton straw on performance of dairy goats (*n* = 10).

Items	CK 1	CK 2	YL-11	*p*-Value
IBW(kg)	48.35 ± 5.17	48.62 ± 4.89	48.72 ± 7.47	0.985
FBW(kg)	53.58 ± 6.79	54.29 ± 7.32	54.32 ± 6.17	0.963
ADG (kg/d)	0.09 ± 0.05	0.09 ± 0.05	0.09 ± 0.05	0.941
DMI (kg/d)	1.64 ± 0.92	1.49 ± 0.66	1.61 ± 0.95	0.920
ADMY (kg/d)	0.48 ± 0.10 ^Bb^	0.35 ± 0.12 ^Cc^	0.57 ± 0.09 ^Aa^	<0.001
4% FCMY (kg/d)	0.51 ± 0.15 ^Bb^	0.37 ± 0.13 ^Bb^	0.72 ± 0.22 ^Aa^	0.001
FMR	3.41 ± 2.05 ^Bb^	4.25 ± 0.45 ^Aa^	2.82 ± 0.83 ^Bb^	<0.001

Data are presented as mean ± SD. Within the same row, significant differences (*p* < 0.05) are denoted by different lowercase letters (^a, b, c^), and extremely significant differences (*p* < 0.01) are denoted by different uppercase letters (^A, B, C^). IBW, initial body weight; FBW, final body weight; ADG, average daily gain; DMI, dry matter intake; ADMY, average daily milk yield;4 % FCMY, 4% fat-corrected milk yield; FMR, feed-to-milk ratio.

**Table 6:** Influence of YL-11 fermented cotton straw on apparent digestibility in dairy goats (*n* = 10, %).

Items	CK 1	CK 2	YL-11	*p*-Value
DM	74.71 ± 4.83 ^a^	68.63 ± 4.76 ^b^	69.13 ± 3.68 ^b^	0.014
EE	61.90 ± 8.12	57.92 ± 5.21	59.07 ± 6.42	0.594
CP	55.21 ± 7.21 ^Aa^	43.02 ± 3.54 ^Bb^	52.46 ± 4.85 ^Aa^	<0.001
NDF	60.10 ± 7.41 ^Aa^	49.12 ± 4.60 ^Bb^	51.16 ± 8.18 ^ABab^	0.006
ADF	44.75 ± 9.45	47.97 ± 7.20	51.46 ± 9.17	0.361
Ash	25.12 ± 9.70 ^Aa^	11.14 ± 3.20 ^Bb^	29.44 ± 5.61 ^Aa^	<0.001

Data are expressed as mean ± SD. Within the same row, different lowercase letters (^a, b^) denote significant differences (*p* < 0.05), whereas different uppercase letters (^A, B^) indicate extremely significant differences (*p* < 0.01). DM, dry matter; EE, ether extract; CP, crude protein; NDF, neutral detergent fiber; ADF, acid detergent fiber.

**Table 7:** Influence of YL-11 fermented cotton straw on milk quality of dairy goats (*n* = 10, %).

Items	CK 1	CK 2	YL-11	*p*-Value
Milk fat percentage	4.41 ± 0.99 ^Bb^	4.30 ± 0.55 ^Bb^	5.77 ± 1.30 ^Aa^	0.004
Milk protein percentage	4.57 ± 1.28	4.97 ± 1.04	5.18 ± 1.34	0.533
Lactose percentage	4.15 ± 0.72 ^b^	4.00 ± 0.54 ^b^	4.67 ± 0.27 ^a^	0.028
Non-fat milk solids	9.42 ± 1.01	9.67 ± 1.00	9.77 ± 0.86	0.706

Values are expressed as mean ± SD. Within the same row, different lowercase letters (^a, b^) denote significant differences (*p* < 0.05), whereas different uppercase letters (^A, B^) indicate extremely significant differences (*p* < 0.01).

**Table 8:** Effect of YL-11 fermented cotton straw on penicillin content in milk of dairy goats (*n* = 10).

Items	CK1	CK2	YL-11
Patulin (μg/kg)	BLQ	BLQ	BLQ ^1^
Penicillin G (μg/kg)	BLQ	BLQ	BLQ

^1^ BLQ indicates that the detection value is below the lower limit of quantification or that the target analyte is not detected in the sample.

**Table 9:** Influence of YL-11 fermented cotton straw on antioxidant and immune parameters in dairy goats (*n* = 10).

Items	CK 1	CK 2	YL-11	*p*-Value
T-AOC (U/mL)	8.53 ± 0.49 ^Bb^	8.30 ± 0.69 ^Bb^	9.36 ± 0.42 ^Aa^	<0.001
SOD (U/mL)	79.49 ± 5.02 ^Bb^	75.24 ± 3.01 ^Bb^	90.57 ± 5.84 ^Aa^	<0.001
CAT (U/mL)	42.49 ± 2.10 ^Bb^	40.69 ± 2.59 ^Bb^	47.97 ± 2.51 ^Aa^	<0.001
GSH-Px (U/mL)	154.07 ± 8.33 ^Bb^	147.77 ± 9.52 ^Bb^	170.26 ± 7.43 ^Aa^	<0.001
MDA (nmol/mL)	3.22 ± 0.58 ^ab^	3.68 ± 0.81 ^a^	2.85 ± 0.43 ^b^	0.022
TNF-α (pg/mL)	46.16 ± 4.62 ^ab^	49.00 ± 5.23 ^a^	43.32 ± 3.05 ^b^	0.027
IL-1β (pg/mL)	20.06 ± 1.35 ^ABab^	21.68 ± 1.64 ^Aa^	17.78 ± 1.07 ^Bb^	<0.001
TGF-β1 (pg/mL)	13.20 ± 0.69 ^Bb^	14.19 ± 0.96 ^Aa^	10.60 ± 0.71 ^Cc^	<0.001
IL-10 (pg/mL)	13.85 ± 1.67 ^Bb^	14.37 ± 1.38 ^ABab^	16.08 ± 1.43 ^Aa^	0.007

Data are shown as mean ± SD. For a given row, significant differences (*p* < 0.05) are marked by different lowercase letters (^a, b, c^), while extremely significant differences (*p* < 0.01) are marked by different uppercase letters (^A, B, C^). The abbreviations are as follows: T-AOC, total antioxidant capacity; SOD, superoxide dismutase; CAT, catalase; GSH-Px, glutathione peroxidase; MDA, malondialdehyde; TNF-α, tumor necrosis factor-α; IL-1β, interleukin-1β; TGF-β1, transforming growth factor-β1; IL-10, interleukin-10.

**Table 10:** Influence of YL-11 fermented cotton straw on serum biochemical parameters in dairy goats (*n* = 10).

Items	CK 1	CK 2	YL-11	*p*-Value
TP (g/L)	87.33±5.85Aa	79.97±3.80Bb	88.18±5.68Aa	0.002
TC (mmol/L)	2.24±0.29	2.28±0.38	1.99±0.41	0.168
TG (mmol/L)	0.24±0.12	0.25±0.08	0.20±0.06	0.350
ALB (g/L)	27.66±2.60Aa	27.38±2.80Aa	24.24±1.66Bb	0.006
GLB (g/L)	59.66±5.18Aa	52.59±3.16Bb	63.94±5.64Aa	<0.001
BUN (mg/dL)	20.99±2.88	21.34±3.02	19.04±1.38	0.111
GLU (mmol/L)	2.20±0.29	1.79±0.42	2.06±0.41	0.070
AST (U/L)	113.66±17.96	105.30±14.86	121.18±32.16	0.317
ALT (U/L)	25.07±7.20	28.61±8.08	27.51±4.64	0.501
ALP (U/L)	72.77±14.90a	51.99±11.53b	52.94±12.88b	0.041

Data are expressed as mean ± SD. Within the same row, significant differences (*p* < 0.05) are denoted by different lowercase letters (^a, b^), whereas extremely significant differences (*p* < 0.01) are denoted by different uppercase letters (^A, B^). The abbreviations are defined as follows: TP for total protein, TC for total cholesterol, TG for triglyceride, ALB for albumin, GLB for globulin, BUN for blood urea nitrogen, GLU for glucose, AST for aspartate aminotransferase, ALT for alanine aminotransferase, and ALP for alkaline phosphatase.

**Table 11:** Influence of YL-11 fermented cotton straw on ruminal fermentation parameters in dairy goats (*n* = 6).

Items	CK 1	CK 2	YL-11	*p*-Value
pH	6.10 ± 0.06 ^Bb^	6.28 ± 0.12 ^Aa^	6.15 ± 0.09 ^Bb^	0.004
NH_3_-N (mg/dL)	13.72 ± 2.55 ^b^	13.18 ± 1.66 ^b^	16.75 ± 3.00 ^a^	0.018
Acetate(mmol/L)	32.79 ± 8.13	29.45 ± 6.46	34.59 ± 5.76	0.333
Propionate(mmol/L)	8.03 ± 1.27	7.84 ± 2.01	7.91 ± 0.93	0.966
Butyrate(mmol/L)	4.86 ± 0.74	4.49 ± 1.29	4.77 ± 0.54	0.701
Isobutyrate(mmol/L)	0.81 ± 0.10	0.72 ± 0.20	0.77 ± 0.11	0.446
Valerate(mmol/L)	0.36 ± 0.05 ^a^	0.27 ± 0.08 ^b^	0.30 ± 0.03 ^ab^	0.024
Isovalerate(mmol/L)	1.02 ± 0.16	0.84 ± 0.24	0.98 ± 0.13	0.132
TVFA (mmol/L)	47.90 ± 9.50	43.63 ± 9.58	49.35 ± 6.98	0.414
Acetate/Propionate	4.04 ± 0.35 ^a^	3.81 ± 0.43 ^b^	4.36 ± 0.32 ^a^	0.025

Data are expressed as mean ± SD. In the same row, significant differences (*p* < 0.05) are denoted by different lowercase letters (^a, b^), while extremely significant differences (*p* < 0.01) are denoted by different uppercase letters (^A, B^). NH_3_-N, ammonia nitrogen; TVFA, total volatile fatty acids.

## Data Availability

The raw 16S rRNA gene sequencing datasets generated and analyzed during the current study are available in the NCBI Sequence Read Archive (SRA) repository, under BioProject accession number PRJNA1453688 (persistent link: https://www.ncbi.nlm.nih.gov/bioproject/PRJNA1453688, accessed on 1 March 2026). The original contributions presented in this study are included in this article. Further inquiries can be directed to the corresponding authors.

## Abbreviations

The abbreviations adopted in this manuscript are listed below:

4%FCM	4% Fat-Corrected Milk
ADF	Acid Detergent Fiber
ADG	Average Daily Gain
ADL	Acid Detergent Liber
ALP	Alkaline Phosphatase
ALT	Alanine Aminotransferase
AST	Aspartate Aminotransferase
BUN	Blood Urea Nitrogen
BW	Body Weight
CAT	Catalase
CP	Crude Protein
DM	Dry Matter
DMI	Dry Matter Intake
EE	Ether Extract
FBW	Final Body Weight
GLU	Glucose
GSH-Px	Glutathione Peroxidase
LEfSe	Linear Discriminant Analysis Effect Size
NDF	Neutral Detergent Fiber
NH_3_-N	Ammonia Nitrogen
OTU	Operational Taxonomic Unit
SOD	Superoxide Dismutase
SCFAs	Short-Chain Fatty Acids
PCoA	Principal Coordinate Analysis
PLS-DA	Partial Least Squares Discriminant Analysis
T-AOC	Total Antioxidant Capacity
TC	Total Cholesterol
TGF-β1	Transforming Growth Factor-β1
TNF-α	Tumor Necrosis Factor-α
TP	Total Protein
VFA	Volatile Fatty Acid

## References

[1] Li R., Zhang S., Liu Q., Wang B., Mi S., Wang C., Chen X., He K., Lv Y., Gao X. (2026). Effects of cotton straw and apple pomace mixed ensilage on growth performance, slaughter performance, meat quality, rumen microbiota and metabolome of Xinjiang Brown cattle. Front. Vet. Sci..

[2] Zhou S., Zhu Z., Chen X., Pu Q., Liu S., Luo Y., Shao Y., Sun Y., Sun X., Shang C. (2025). Study on the effects of urea addition on the fermentation quality, nitrogen metabolism, microbial community, and metabolic characteristics of cotton strawlage. Front. Microbiol..

[3] Yue Y., Lin J., Lv G., Liu B., Deng X., Li Y., Li X., Chen K. (2025). Effects of replacing soybean meal with cottonseed meal in amino acid balanced diets on growth performance, apparent digestibility, ruminal fermentation, and microbial diversity in fattening Dorper × Hu crossbred dairy goat. Front. Vet. Sci..

[4] Rashid G.M.M., Bugg T.D.H. (2021). Enhanced biocatalytic degradation of lignin using combinations of lignin-degrading enzymes and accessory enzymes. Catal. Sci. Technol..

[5] Mei J., Shen X., Gang L., Xu H., Wu F., Sheng L. (2020). A novel lignin degradation bacteria-*Bacillus amyloliquefaciens* SL-7 used to degrade straw lignin efficiently. Bioresour. Technol..

[6] Wang L., Wang J., Wang P., Liu C., Li X., Chang J., Jin S., Yin Q., Zhu Q. (2024). Effect of corn straw treated with Lactobacillus plantarum and cellulase on ruminal fermentation and microbiota of Hu dairy goat. Fermentation.

[7] Hu R., Wu D., Liang X., Wang Z., Zou H., Wu F., Li H., Jiang Y., Peng Q., Xiao J. (2025). Solid state fermentation improves the utilization value of cotton stalk. Ind. Crops Prod..

[8] Zhao X., Wang F., Fang Y., Zhou D.W., Wang S.P., Wu D.Q., Wang L.X., Zhong R.Z. (2020). High-potency white-rot fungal strains and duration of fermentation to optimize corn straw as ruminant feed. Bioresour. Technol..

[9] Wu K., Sun X., Xu J., Guan Z., Yuan W., Bao L., Zhao Y., Shan R., Chen H., Zhao C. (2025). Yeast-fermented feed improves high-concentrate diet-induced mastitis in dairy goats by regulating rumen microbiota. Front. Microbiol..

[10] Li J., Luo D., Yang B., Liang X., He X., Zhou G., Wang Z., Zhang K., Liu G., Yang Y. (2025). Effects of probiotic-fermented milk thistle meal on dairy goat production, milk quality, serum biochemistry, and rumen-fecal metagenomes. Food Biosci..

[11] Datsomor O., Gou-Qi Z., Miao L. (2022). Effect of ligninolytic axenic and coculture white-rot fungi on rice straw chemical composition and in vitro fermentation characteristics. Sci. Rep..

[12] Dashora K., Gattupalli M., Javed Z., Tripathi G.D., Sharma R., Mishra M., Bhargava A., Srivastava S. (2022). Leveraging multiomics approaches for producing lignocellulose degrading enzymes. Cell. Mol. Life Sci..

[13] Zhao J., Mu Y., Gong P., Liu B., Zhang F., Zhu L., Shi C., Lv X., Luo J. (2024). Whole-genome resequencing of native and imported dairy goat identifies genes associated with productivity and immunity. Front. Vet. Sci..

[14] Rehemujiang H., Yimamu A., Wang Y.L. (2019). Effect of dietary cotton stalk on nitrogen and free gossypol metabolism in dairy goat. Anim. Biosci..

[15] (2007). Nutrient Requirements of Small Ruminants: Sheep, Goats, Cervids, and New World Camelids.

[16] Piantoni P., Lock A.L., Allen M.S. (2013). Palmitic acid increased yields of milk and milk fat and nutrient digestibility across production level of lactating cows. J. Dairy Sci..

[17] Zhou J., An J., Dong C., Zhang Y., Zhao Q., Cui Y., Jiao B. (2020). Dissipation and residue determination of penicillin G and its two metabolites in citrus under field conditions by DSPE/UPLC-MS/MS. Biomed. Chromatogr..

[18] Tannous J., Atoui A., El Khoury A., Kantar S., Chdid N., Oswald I.P., Puel O., Lteif R. (2015). Development of a real-time PCR assay for *Penicillium expansum* quantification and patulin estimation in apples. Food Microbiol..

[19] William H.G.W. (2006). Official Methods of Analysis of AOAC International. Official Methods of Analysis of AOAC International UK.

[20] Van Soest P.J., Robertson J.B., Lewis B.A. (1991). Methods for dietary fiber, neutral detergent fiber, and nonstarch polysaccharides in relation to animal nutrition. J. Dairy Sci..

[21] Panche A.N., Diwan A.D., Chandra S.R. (2016). Flavonoids: An overview. J. Nutr. Sci..

[22] Bampidis V., Azimonti G., Bastos M.L., Christensen H., Dusemund B., Durjava M.K., Kouba M., López-Alonso M., Puente S.L., EFSA Panel on Additives and Products or Substances used in Animal Feed (FEEDAP) (2020). Safety and efficacy of Panavital feed (d-glyceric acid) for chickens for fattening. EFSA J..

[23] Jin Y., An H.J., Zheng T.T., Li J.J., Gao J.M., Zhong X.L., Li B.H., Liu Y.Y., Zhuang X.J., Chen J.H. (2026). Adenosine from high-fat-diet-tolerant monkey-derived Limsolactobacillus reuteri MacFasB02 modulates cholesterol metabolism to alleviate hyperlipidemia and inflammation. npj Sci. Food.

[24] Behbahani A.B., Ghasemi P., Vasiee A., Mousavi Z. (2026). Probiotic, functional, and safety assessment of Levilactobacillus brevis M3R3 isolated from traditional Khiki cheese. Antonie Leeuwenhoek.

[25] Mihalchik L.A., Roberts A., LaCombe J., Lloyd M., Doepker C., Eapen A. (2026). Evaluation of the safety and potential genotoxicity of a high-purity mogroside ingredient containing siamenoside I produced from a modified strain of Yarrowia lipolytica. Toxicol. Rep..

[26] Vinatzer B.A., van Zonneveld M., Ramirez M., Williams D.E., Petz M., Meckelmann S., Avila T., Bejarano C., Ríos L., Peña K. (2015). Screening genetic resources of Capsicum peppers in their primary center of diversity in Bolivia and Peru. PLoS ONE.

[27] Liu Q., Zhang X., Li X., Li J., Guo L., Zhong W. (2025). Effects of fermentation duration on nutritional value, probiotic components and growth performance in beef cattle fed fermented Chinese medicine residue. J. Appl. Anim. Res..

[28] Wang D., Chen L., Tang G., Yu J., Chen J., Li Z., Cao Y., Lei X., Deng L., Wu S. (2023). Multi-omics revealed the long-term effect of ruminal keystone bacteria and the microbial metabolome on lactation performance in adult dairy goats. Microbiome.

[29] Li X.Z., Park B.K., Yan C.G., Choi J.G., Ahn J.S., Shin J.S. (2012). Effect of alcohol fermented feed on lactating performance, blood metabolites, milk fatty acid profile and cholesterol content in holstein lactating cows. Anim. Biosci..

[30] Zheng C., Cone J.W., van Peer A., Baars J.J.P., Hendriks W.H. (2025). Conversion of lignocellulosic biomass into valuable feed for ruminants using white rot fungi. J. Anim. Physiol. Anim. Nutr..

[31] Grant R.J., Ferraretto L.F. (2018). Silage review: Silage feeding management: Silage characteristics and dairy cow feeding behavior. J. Dairy Sci..

[32] Yu L., An Z., Xie D., Yin D., Xie G., Gao X., Xiao Y., Liu J., Fang Z. (2024). From waste to protein: A new strategy of converting composted distilled grain wastes into animal feed. Front. Microbiol..

[33] Gule A.H., Derese D.B., Erge C.M., Girgo U.G., Ejeta H.K. (2023). Effect of dietary supplementation of duckweed on growth performance, carcass and non-carcass traits of horro rams fed on a commercial-based diet. Heliyon.

[34] Takiya C.S., Chesini R.G., de Freitas A.C., Grigoletto N.T.S., Vieira D.J.C., Poletti G., Martins N.P., Sbaralho O.P., Roth N., Acedo T. (2024). Dietary supplementation with live or autolyzed yeast: Effects on performance, nutrient digestibility, and ruminal fermentation in dairy cows. J. Dairy Sci..

[35] Amin A.B., Zhang L., Zhang J., Mao S. (2022). Fermented soybean meal modified the rumen microbiome to enhance the yield of milk components in Holstein cows. Appl. Microbiol. Biotechnol..

[36] Sun X., Wang Y., Wang E., Zhang S., Wang Q., Zhang Y., Wang Y., Cao Z., Yang H., Wang W. (2021). Effects of *Saccharomyces cerevisiae* culture on ruminal fermentation, blood metabolism, and performance of high-yield dairy cows. Animals.

[37] Buckhaus E.M., Smith Z.K. (2021). PSIV-1 Effect of corn silage inclusion rate on apparent total tract digestibility of dry matter, organic matter, and crude protein in finishing steers. J. Anim. Sci..

[38] Anh N.M., Minh L.T.H., Linh N.T., Dao P.T., Quynh D.T., Huong D.T.M., Van Cuong P., Huyen V.T.T., Dat T.T.H. (2024). Secondary metabolites from marine fungus *Penicillium chrysogenum* VH17 and their antimicrobial and cytotoxic potential. Biosci. Biosci. Biotechnol. Biochem..

[39] Shao P., Sha Y., Liu X., He Y., Wang F., Hu J., Wang J., Li S., Chen X., Yang W. (2024). Supplementation with Astragalus Root Powder Promotes Rumen Microbiota Density and Metabolome Interactions in Lambs. Animals.

[40] Juárez-Cisneros G., Campos-García J., Díaz-Pérez S.P., Lara-Romero J., Tiwari D.K., Sánchez-Yáñez J.M., la Cruz H.R.-D., Jiménez-Sandoval S., Villegas J. (2021). Ligninolytic activity of the *Penicillium chrysogenum* and *Pleurotus ostreatus* fungi involved in the biotransformation of synthetic multi-walled carbon nanotubes modify its toxicity. PeerJ.

[41] Glover J., Duthie D.W. (1958). The apparent digestibility of crude protein by non ruminants and ruminants. J. Agric. Sci..

[42] Zhou R., Wang L., Li Y., Wu H., Lu L., Zang R., Xu H. (2024). Effects of tail vegetable fermented feed on the growth and rumen microbiota of lambs. Animals.

[43] Wang C., Wei S., Xu B., Hao L., Su W., Jin M., Wang Y. (2021). *Bacillus subtilis* and *Enterococcus faecium* co-fermented feed regulates lactating sow’s performance, immune status and gut microbiota. Microb. Biotechnol..

[44] He S., Zhang R., Wang R., Wu D., Dai S., Wang Z., Chen T., Mao H., Li Q. (2024). Responses of nutrient utilization, rumen fermentation and microorganisms to different roughage of dairy buffaloes. BMC Microbiol..

[45] Wu X., Dhanasekaran S. (2020). Protective effect of leaf extract of *Abutilon indicum* on DNA damage and peripheral blood lymphocytes in combating the oxidative stress. Saudi Pharm. J..

[46] Lee M.T., Lin W.C., Yu B., Lee T.T. (2017). Antioxidant capacity of phytochemicals and their potential effects on oxidative status in animals—A review. Anim. Biosci..

[47] Wang W.K., Li W.J., Wu Q.C., Wang Y.L., Li S.L., Yang H.J. (2021). Isolation and identification of a rumen Lactobacillus bacteria and its degradation potential of gossypol in cottonseed meal during solid-state fermentation. Microorganisms.

[48] Li J., Gao T., Hao Z., Guo X., Zhu B. (2022). Anaerobic solid-state fermentation with Bacillus subtilis for digesting free gossypol and improving nutritional quality in cottonseed meal. Front. Nutr..

[49] Hine B.C., Hunt P.W., Colditz I.G. (2019). Production and active transport of immunoglobulins within the ruminant mammary gland. Vet. Immunol. Immunopathol..

[50] Jin D., Zhao S., Zheng N., Beckers Y., Wang J. (2018). Urea metabolism and regulation by rumen bacterial urease in ruminants—A review. Ann. Anim. Sci..

[51] Sun F., Aguerre M.J., Wattiaux M.A. (2019). Starch and dextrose at 2 levels of rumen-degradable protein in iso-nitrogenous diets: Effects on lactation performance, ruminal measurements, methane emission, digestibility, and nitrogen balance of dairy cows. J. Dairy Sci..

[52] Roffler R.E., Satter L.D. (1975). Relationship between ruminal ammonia and nonprotein nitrogen utilization by ruminants. II. Application of published evidence to the development of theoretical model for predicting nonprotein nitrogen utilization. J. Dairy Sci..

[53] Huang K., Chen H., Liu Y., Hong Q., Yang B., Wang J. (2022). Lactic acid bacteria strains selected from fermented total mixed rations improve ensiling and in vitro rumen fermentation characteristics of corn stover silage. Anim. Biosci..

[54] Azizi A., Jolazadeh A., Sharifi A., Khalifeh M.J. (2026). Dietary substitution of conventional forage resources with camelina seed pods as a novel feed ingredient on rumen parameters, blood metabolites, microbial protein synthesis, nutrient intake, growth performance and behavioral responses in fattening lambs. Anim. Feed Sci. Technol..

[55] Ashkvari A., Rezaei J., Fazaeli H., Dehghan S.A. (2026). Effect of new multistrain Bacilli, Lactobacilli, yeast, or their mixtures on in vitro ruminal microbial populations, hydrolytic enzymes, and fermentation variables of dairy goat. Anim. Feed Sci. Technol..

[56] Gomes B.M., Coelho S.G., Neves L.F.M., Souza E.M.B., Campolina J.P., Diavão J., Silva A.S., Tomich T.R., Carvalho W.A., Campos M.M. (2026). Effect of thermal amplitude on physiological parameters, ruminal fermentation, digestibility, health, and performance of Holstein dairy calves. J. Dairy Sci..

[57] Guo X., Li Z., Lin X., Pei Y., Wang Z., Ma Y., Li Y., Xu H., Ma F., Shen Y. (2025). Ruminal degradation of puerarin and its effects on in vitro ruminal fermentation, methane emission, and microbial community structure. Animals.

[58] Lou X., Malik I.M., Yang Y., Huang Y., Zhao X., Zhang Q., Xu Z., Wang J., Zhang S., Zhao G. (2026). Effects of fermented total mixed diets on growth performance, nutrient digestibility, serum indicators, rumen fermentation and rumen microbiota in Tibetan dairy goat. Anim. Feed Sci. Technol..

[59] Musati M., Coppa M., Delbes C., Verdier-Metz I., Popova M., Niderkorn V., Bouchon M., Farizon Y., Enjalbert F., Renna M. (2025). The ruminal and faecal microbiota, digestion processes, and milk composition of dairy cows are modified by the botanical biodiversity of pastures. Animal.

[60] Dong D., Yao H., Aihemaiti M., Ainizirehong G., Li Y., Yan Y., Huang X., Hou M., Cui W. (2025). Enhanced nutritional composition of steam-exploded cotton stalk through microbial-enzyme synergism solid-state fermentation. Fermentation.

